# Lip cyanosis as the first symptom of Leigh syndrome associated with mitochondrial complex I deficiency due to a compound heterozygous *NDUFS1* mutation: A case report

**DOI:** 10.1097/MD.0000000000030303

**Published:** 2022-08-26

**Authors:** Lina Men, Jinxing Feng, Weimin Huang, Mingguo Xu, Xiaoli Zhao, Ruixin Sun, Jianfang Xu, Liming Cao

**Affiliations:** a Department of Neurology, Shenzhen Children’s Hospital, Shenzhen, China; b Department of Pediatric, the Third People’s Hospital of Longgang District, Shenzhen, China; c Department of Neurology, The First Affiliated Hospital of Shenzhen University, Shenzhen, China.

**Keywords:** case report, cyanosis, Leigh syndrome, mitochondrial disease, *NDUFS1* gene

## Abstract

Leigh syndrome (LS) is a rare, progressive, and fatal neurodegenerative disease that occurs mainly in infants and children. Neonatal LS has not yet been fully described.

**Methods:**

The study design was approved by the ethics review board of Shenzhen Children’s Hospital.

**Results:**

A 24-day-old full-term male infant presented with a 2-day history of lip cyanosis when crying in September 2021. He was born to nonconsanguineous Asian parents. After birth, the patient was fed poorly. A recurrent decrease in peripheral oxygen saturation and difficulty in weaning from mechanical ventilation during hospitalization were observed. There were no abnormalities on brain magnetic resonance imaging (MRI) or blood and urine organic acid analyses on admission. His lactic acid level increased markedly, and repeat MRI showed symmetrical abnormal signal areas in the bilateral basal ganglia and brainstem with disease progression. Trio whole-exome sequencing revealed 2 heterozygous mutations (c.64C > T [p.R22X] and c.584T > C [p.L195S]) in *NDUFS1*. Based on these findings, mitochondrial respiratory chain complex I deficiency-related LS was diagnosed. The patient underwent tracheal intubation and mechanical ventilation for respiratory failure. His oxygen saturation levels were maintained at normal levels with partially assisted ventilation. He was administered broad-spectrum antibiotics, oral coenzyme Q10, multivitamins, and idebenone. During hospitalization, the patient developed progressive consciousness impairment and respiratory and circulatory failure. He died on day 30.

**Conclusion:**

Lip cyanosis is an important initial symptom in LS. Mild upper respiratory tract infections can induce LS and aggravate the disease. No abnormal changes in the brain MRI were observed in the early LS stages in this patient. Multiple MRIs and blood lactic acid tests during disease progression and genetic testing are important for prompt and accurate diagnosis of LS.

## 1. Introduction

Leigh syndrome (LS), a mitochondrial encephalomyopathy caused by mutations in the mitochondrial DNA (mtDNA) or nuclear DNA (nDNA), is a fatal, progressive neurodegenerative disease.^[1–3]^ The estimated incidence rate of LS is 1:40,000 live births.^[1,3]^ Neuropathological characteristics of LS include bilateral symmetrical lesions, particularly in the basal ganglia and brainstem regions. The clinical features include psychomotor retardation, respiratory difficulties, nystagmus, ophthalmoparesis, optic atrophy, ataxia, and dystonia.^[4]^

LS has a Mendelian inheritance pattern and is caused by >60 identified mutations in the mtDNA and nDNA.^[5]^ Whole-exome sequencing (WES) has greatly improved genetic diagnosis for highly suspected cases of mitochondrial disorders. However, WES does not produce an effortless diagnosis in all cases because the data may not provide sufficient certainty for a definitive diagnosis and there is a waiting period for WES results. In patients with complicated and fast-evolving LS, bedside diagnosis and rapid treatment still require mastering the clinical characteristics. Mutations in genes encoding nuclear-encoded subunits of complex I of the mitochondrial respiratory chain (MRC) are recognized causes of LS.^[4]^ The clinical features of neonatal-type LS have not yet been fully elucidated. Herein, we present such a case and review the literature to improve diagnosis.

## 2. Methods

The study design was approved by the ethics review board of Shenzhen Children’s Hospital (No: 2022-004).

## 3. Case presentation

A 24-day-old full-term male infant presented with lip cyanosis without inspiratory dyspnea while crying for 2 days in September 2021. He was born via cesarean section, with a birth weight of 2.85 kg and APGAR scores of 10 at 1 minute and 5 minutes (normal) at birth. He had no history of perinatal infection or asphyxia. His parents were healthy and nonconsanguineous Asian parents. He was the third child of 3 children; he had a healthy 8-year-old sister and a sister who died in infancy. The patient was fed poorly after birth. Physical examination on admission revealed a pulse rate of 164 beats per minute, respiratory rate of 32 breaths per minute, blood pressure of 70/41 mm Hg, weight of 3.55 kg, clear consciousness, slight irritation, cyanosis on crying, bilateral coarse breath sounds with mild moist rales, regular heart rhythm, normal limb muscle tone, and normal reflexes (sucking, rooting, and Moro reflexes). Furthermore, his peripheral oxygen saturation (SpO_2_) was 85%, which was maintained at approximately 90% with mask oxygen therapy.

**Figure 1.**
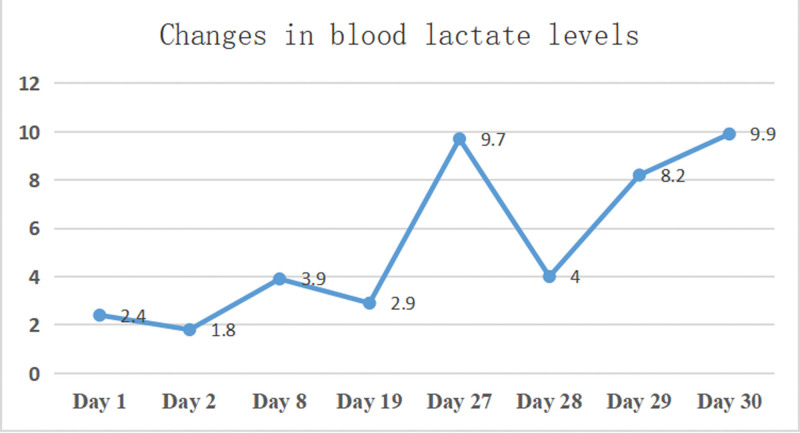
Changes in blood lactate levels in the course of the disease.

His initial blood analyses revealed white blood cell count, C-reactive protein, creatinine, and procalcitonin levels within the normal ranges; decreased hemoglobin level (111, reference range: 170–200 g/L), increased platelet count (473, reference range: 100–300 × 10^9^/L), and slightly elevated lactate level (2.4, reference range: 1–1.8 mmol/L) at admission. Repeat testing revealed slowly progressive blood lactate levels (Fig. 1). The cytomegalovirus IgG antibody titer was elevated (52.1, reference range: 0–14 U/mL). Tests for cytomegalovirus DNA in the blood and severe acute respiratory syndrome coronavirus 2 (SARS-CoV-2) nucleic acid were negative. The initial arterial blood gas analysis showed a normal pH, decreased oxygen partial pressure (39, reference range: 83–108 mm Hg) and oxygen saturation (85%, reference range: 94–98%), and increased standard bicarbonate concentration (25.7, reference range: 22–27 mmol/L) and carbon dioxide partial pressure (63; reference range: 35–45 mm Hg).

**Figure 2.**
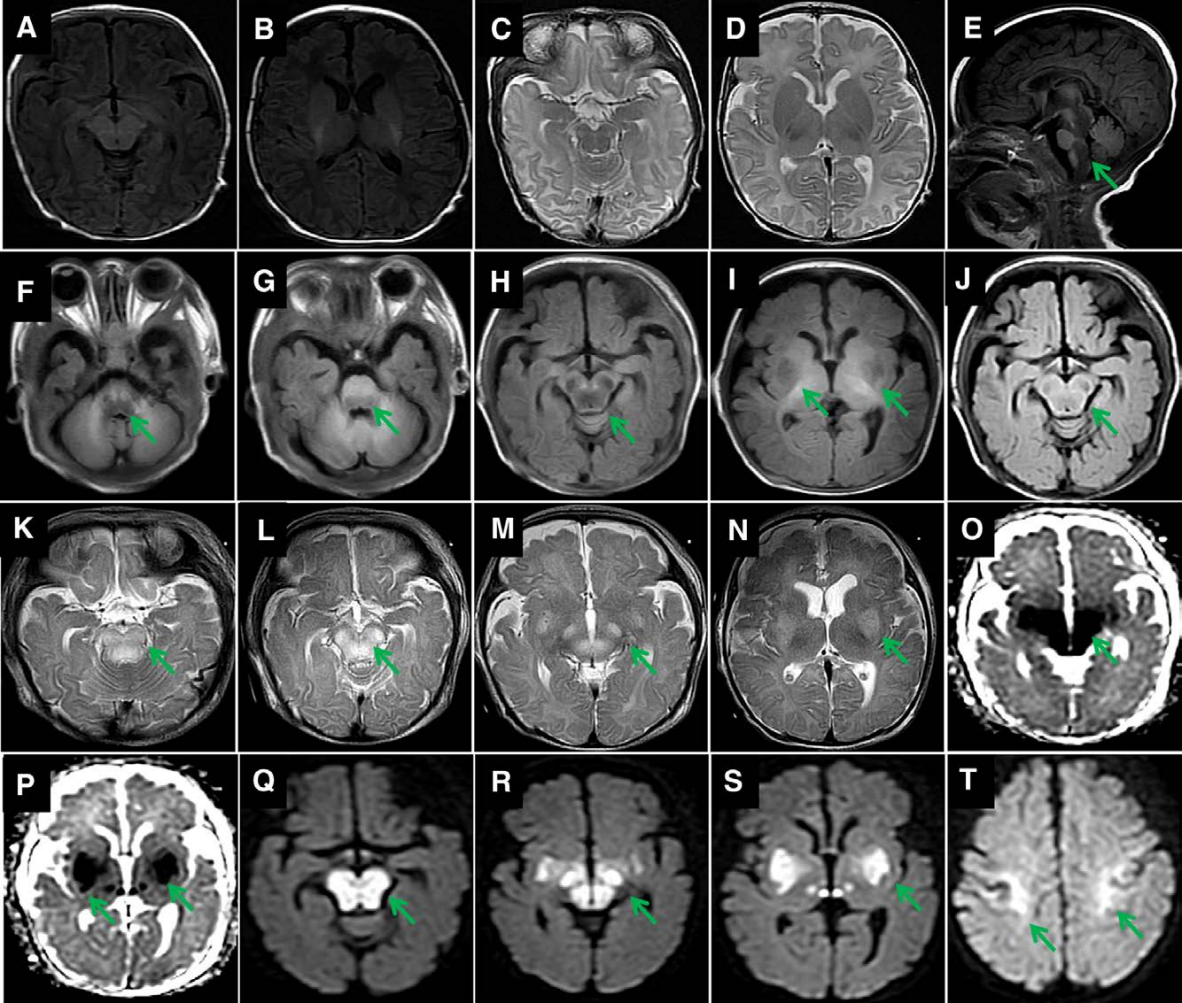
Evolution of lesions on MRI. Brain MRI (A, B, C, and D) on day 8 showing no obvious abnormalities. MRI on day 30 showing symmetrical lesions in the bilateral basal ganglia, cerebral peduncle, and brainstem hypointense on T1-WI (E, F, G, H, and I, arrows), fluid-attenuated inversion recovery imaging (J, arrow), apparent diffusion coefficient (O and P, arrows), hyperintensity on T2-WI (K, L, M and N, arrows), and diffusion limitation on diffusion WI (Q, R, S and T, arrows). MRI = magnetic resonance imaging, WI = weighted imaging.

**Figure 3.**
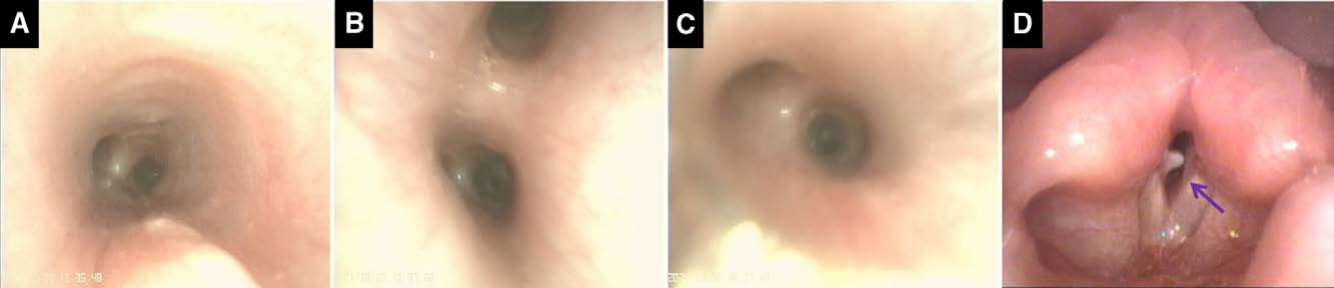
Laryngoscopy and fiberoptic bronchoscopy findings. Fiberoptic bronchoscopy showing congestion and edema of the tracheal mucosa, sharp carina, no expiratory tracheal collapse, or stenosis during the expiratory phase (A). The bronchial segment opening of the left (B) and right lung (C) is basically normal. Laryngoscopy showing granulation-like protrusion on the right posterior vocal cord (D, arrow) and bilateral incomplete closure of the vocal cord.

A routine cerebrospinal fluid (CSF) analysis revealed that CSF protein levels were within normal limits. The CSF culture was negative. Spectral analysis of amino acids and acylcarnitine in the blood showed no apparent abnormalities. On day 2, chest radiography revealed bilateral lung markings and atelectasis in the right upper lung. Echocardiography revealed a patent foramen ovale and mild tricuspid regurgitation. On day 8, brain magnetic resonance imaging (MRI) showed no apparent abnormalities (Fig. 2A–D). However, on day 30, MRI revealed symmetrical lesions in the bilateral basal ganglia, cerebral peduncle, and brainstem with low signal on T1-weighted imaging (WI, Fig. 2E–I), fluid-attenuated inversion recovery imaging (Fig. 2J), apparent diffusion coefficient (Fig. 2O and P), high signal on T2-WI (Fig. 2K–N), and diffusion limitation on diffusion-WI (Fig. 2Q–T). 24-h dynamic electrocardiography showed paroxysmal atrial tachycardia and sporadic atrial extrasystoles, and amplitude-integrated electroencephalography (Fig. 3A) suggested slightly delayed development. The 15-h long-term video-electroencephalography (Fig. 3B) showed no epileptiform waves, especially when his SpO_2_ decreased.

**Figure 4.**
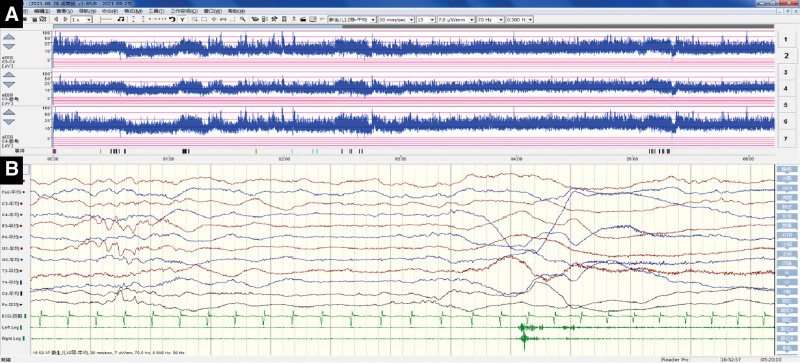
Findings of amplitude-integrated EEG and long-term video EEG. Amplitude-integrated EEG (A) suggests slightly delayed development, and there are no seizure patterns on long-term video EEG (B) when the arterial oxygen saturation decreases. EEG = electroencephalography.

Tracheal intubation was performed, and he was placed on mechanical ventilation on day 1, followed by noninvasive intermittent positive-pressure ventilation, intravenous ceftazidime for 19 days, oral furosemide and spironolactone for 7 days, and sputum suction. Tracheal intubation was administered repeatedly. Furthermore, he was placed on mechanical ventilation 5 times (averagely 3.4 days per time) and noninvasive intermittent positive-pressure ventilation 4 times (averagely 2 days per time) and failed weaning from mechanical ventilation. His SpO_2_ decreased after weaning, especially during sleep but was maintained at normal levels with slightly assisted ventilation. On day 10, the patient had recurrent cyanosis, poor response to stimulation, decreased muscular tension, intermittent nystagmus, and fever. Fiberoptic bronchoscopy revealed no bronchial stenosis or obstruction (Fig. 4A–C). Laryngoscopy (Fig. 4D) revealed a granulation-like protrusion in the right posterior vocal cord and incomplete bilateral closure of the vocal cords on day 17. We suspected that the unexplained hypoxia might be related to the laryngoscopy findings; hence, partial laryngectomy was performed under general anesthesia the following day. The postoperative dyspnea did not improve.

**Figure 5.**
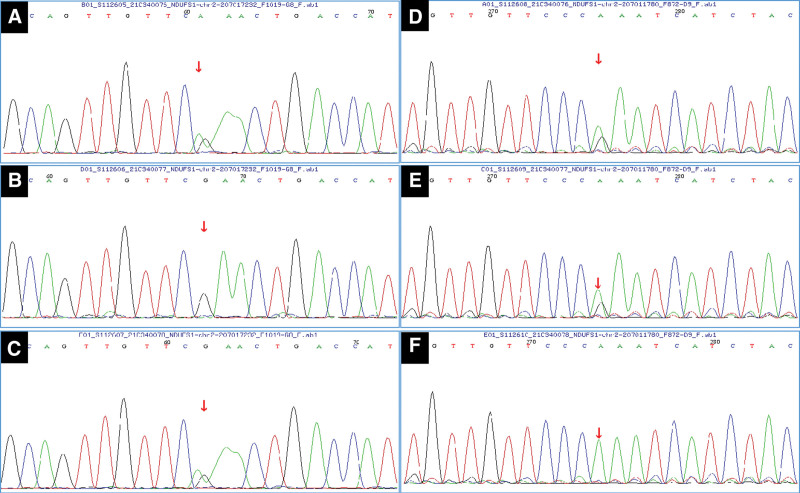
Sanger sequencing of heterotopic sites of *NDUFS1* in families with mitochondrial respiratory chain complex I deficiency nuclear type 5. (A) Heterozygous mutation (c.64C > T, arrow) in exon 3 in the proband, (B) wild-type genotype in the proband father, and (C) mother of the proband has the same heterozygous mutation at the locus (arrow). (D) Heterozygous mutation (c.584T > C, arrow) in exon 8 in the proband, (E) the father of the proband has the same heterozygous mutation (c.584T > C, arrow), and (F) wild-type genotype in the proband mother.

WES and mitochondrial gene sequencing using peripheral blood samples from the children and their parents (Beijing Maijinuo Medical Laboratory, Fig. 5) on day 24 revealed a compound heterozygous mutation (c.64C > T [p.R22X] and c.584T > C [p.L195S]) in *NDUFS1* gene. Based on the genetic testing results, the patient was administered oral levocarnitine, coenzyme Q10, vitamin B1, vitamin B2, vitamin B6, and idebenone. Blood oxygen levels were unstable during invasive assisted ventilation, with fever on day 27. Therefore, he was administered potent broad-spectrum antibiotics (intravenous cefoperazone, meropenem, and vancomycin). However, the patient had progressive loss of consciousness and developed respiratory and circulatory failure for which cardiopulmonary resuscitation was performed, and intravenous adrenaline was administered. Unfortunately, the patient died 30 days after hospitalization.

## 4. Discussion

Here, we describe lip cyanosis as the first symptom of mitochondrial respiratory chain complex I deficiency (MCID)-related LS, caused by a compound heterozygous mutation in *NDUFS1*. The clinical phenotype of our patient was consistent with that of MCID. The patient was diagnosed with LS associated with MCID due to a rare *NDUFS1* mutation. This case report enriches the clinical phenotypic spectrum of neonatal LS and emphasizes that MRI findings may appear normal in the early stages. Repeated respiratory muscle weakness should cause clinicians to suspect MCID-related LS. Reexamination of lactate levels and MRI, especially as the disease progresses, is important in the diagnosis of LS. The patient’s condition progressed rapidly and was likely to be misdiagnosed. Co-segregation analysis confirmed that the mutation in our patient was inherited from the heterozygous carrier parents. To our knowledge, this is the second reported case of MRC complex I deficiency caused by a pathogenic mutation (c.64C > T [p.R22X]) in *NDUFS1*.^[6]^ Precise gene analysis allows clinicians to counsel patients and their families about treatment, the risk of recurrence, prenatal testing, and prognosis.

### 4.1. Leigh syndrome

LS can be subdivided into 3 phenotypes (neonatal, classic infant, and juvenile) according to the age at onset.^[7]^ Neonatal-type LS initially presents with feeding and swallowing disorders, dyspnea, brainstem dysfunction (such as abnormal eye movement behavior and facial muscle weakness), and severe motor development retardation.^[8]^ The first symptoms^[9]^ of LS are dyskinesia (82.8%, including hypotonia, spasticity, ataxia, and chorea), ocular symptoms (25%), feeding or sucking difficulties (14.1%), seizures (13.3%), and growth retardation (10.2%). The most frequently involved systems in progressive LS are the motor, eye, and digestive systems, observed in more than half of the patients.

### 4.2. Diagnosis

The diagnosis of LS is based on clinical observations, family history, laboratory evaluations, imaging, histochemical staining of muscle biopsies, MRC enzyme activity analysis, and identification of mtDNA or nDNA pathogenic variant(s). LS is defined as ① a neurodegenerative disease with variable symptoms, ② mitochondrial dysfunction caused by a hereditary genetic defect, and ③ accompanied by bilateral central nervous system lesions.^[5]^ Our patient met the diagnostic criteria for LS.

### 4.3. Neuroimaging

Bilateral symmetric basal ganglia and/or brainstem lesions are the MRI features of LS.^[10,11]^ Some experts believe that bilateral symmetric putamen involvement is an essential feature of LS,^[8]^ which was verified in our case. Patients with LS do not have a propensity for cerebral white matter or cortical involvement.^[5]^ However, the brainstem and basal ganglia are not involved in few cases.^[12]^ Instead, brain white matter involvement^[8]^ may lead to severe early onset leukoencephalopathies.^[13]^ Bilateral precentral gyrus involvement in diffusion-weighted imaging was observed in this case, which has not been fully reported before. These findings reflect neuroimaging heterogeneity in patients with LS, symmetrical diffusion-limited lesions like those in our patient, and increased lactate peaks on magnetic resonance spectroscopy,^[12]^ which are also important imaging features.

In this case, there was no obvious abnormality in the brain MRI at the initial stage of LS—a key diagnostic feature. Repeat MRI revealed typical imaging changes during aggravation of the disease: symmetrical lesions in the bilateral brainstem and basal ganglia, consistent with the neuroimaging features of LS.^[11]^ This case highlights the importance of repeat MRI and that LS cannot be excluded by MRI negativity in the early disease stage.

### 4.4. Mitochondrial respiratory chain complex I deficiency

Approximately 90% of cell energy requirements are achieved through hydrolysis of adenosine triphosphate (ATP). The leading cause of mitochondrial disease is an oxidative phosphorylation disorder caused by MRC dysfunction, which secondarily causes ATP reduction. The damage may be more serious in tissues and organs with higher energy demands, such as the brain, skeletal muscles, and heart.^[14]^ MRC is composed of 5 enzymatic complexes (I–V). Complex I, also known as CoQ reductase, is the largest enzyme complex and plays an important role in ATP production. Defects in the MRC enzyme complex caused by mitochondrial and nuclear gene mutations can cause LS, and complex I and IV defects are the most common.^[14]^

MCID is a genetically heterogeneous disease. MCID causes a decrease in ATP production and an increase in reactive oxygen species production. Defects in the coding sequences of multiple nuclear genes can lead to 33 subtypes of MCID based on different pathogenicities. Defects in mitochondrial complex I are genetically heterogeneous and clinically associated with a wide range of presentations, including LS, stroke-like episodes (MELAS), and leukodystrophy.^[15,16]^ Most affected individuals present during their first year of life, and the disease has a rapidly progressive and fatal course.^[15,16]^

MCID nuclear type 5 (MC1DN5) is caused by rare mutations in the NADH dehydrogenase Fe-S protein 1-coding *NDUFS1* gene.^[17]^ The onset of symptoms in patients with MCIDN5 is usually in infancy, like in our patient, who was symptomatic and showed signs of mitochondrial dysfunction, brain imaging abnormalities, elevated blood and CSF lactic acid, and defective MRC complex enzyme I activity. MCID has a wide range of clinical presentations, including LS, and its genetic causes are heterogeneous, with poor genotype-phenotype correlations.

Our patient had feeding difficulties, and electroencephalography suggested slightly delayed development. Lip cyanosis is associated with respiratory muscle weakness secondary to medulla oblongata injury. Lip cyanosis may be an early sign of respiratory failure that needs to be considered.

### 4.5. NADH dehydrogenase Fe-S protein 1

NADH dehydrogenase Fe-S protein 1 (NDUFS1) is the largest subunit of complex I. *NDUFS1* encodes one of the 14 highly conserved core subunits of complex I.^[18]^ *NDUFS1* mutations are the most common nDNA abnormalities reported in patients with complex I deficiency.^[15]^ *NDUFS1* is located on chromosome 2q33.3, contains 20 exons, and encodes a protein composed of 727 amino acids.^[6]^ More than 120 *NDUFS1* loci are currently included in the ClinVar database, including 35 missense variants; the remainder are frameshift, nonsense, and shear mutations.^[6]^ The Trio-WES analysis identified compound heterozygous *NDUFS1* variants (maternal c.64C > T [p.R22X] and paternal c.584T > C [p.L195S]) in the proband. The compound heterozygous *NDUFS1* mutation in this proband consisted of a nonsense mutation (p.R22X) inherited from the mother and a missense mutation (p.L195S) inherited from the father. According to the American College of Medical Genetics and Genomics guidelines, c.64C > T and c.584T > C variants in *NDUFS1* of the proband were predicted to be pathogenic variants and variants with uncertain significance, respectively. Although most *NDUFS1* mutations cause severe and rapidly progressive leukoencephalopathy, milder presentations have been reported.^[19]^ The clinical features and outcomes of patients with mutated *NDUFS1*-related MCID are summarized in Table 1.

**Table 1:** Demographics, clinical features, main therapy, and outcomes of patients with respiratory chain complex I deficiency due to mutations in *NDUFS1.*

Study	Age/sex	Genetic history	Neurological symptoms and signs	Brain MRI	Biochemical analysis	Variation	Main therapy	Outcome
Gao et al^[6]^	10-M-old/male	No mention	Slow response, difficulty in turning over and sitting up after fever at 10 mo old, with increased muscle tension and bilateralpositive Babinski sign.	MRI showed multiple abnormal signals and vacuole-like changes around bilateral lateral ventricles and centrum semiovale, without enhancement.	Increased blood lactate levels.	Compound heterozygous (paternal c.64C > T and maternal c.845A > G).	Comprehensive treatment and management.	Motor ability and intellectual development improved.
Hoefs et al^[12]^	No mention of age/female	She was born to healthy, unrelated parents.	She had modest intrauterine growth retardation and started at age 8 mo with abnormal crying and regression of already acquired motor skills.	MRI at 9 mo showed symmetric hyperintensity on T2, hypointensity on T1 in WM sparing U-fibers, and slightly atrophic corpus callosum. MRI at 11 mo showed a progressive WM lesion and corpus callosum atrophy; cerebral cortex, basal ganglia, and brainstem were unaffected	No mention.	Homozygous for c.1855G > A.	No mention.	She ultimately developed spasticity, microcephaly, mental retardation, and progressive neuropathy and died at the age of 12 years.
Hoefs et al^[12]^	No mention of age/male	Consanguineous parents. A brother had a similar clinical presentation.	Leukoencephalopathy, episodic brainstem events, reduced spontaneous movement, and an abnormal breathing.	No mention.	Plasma lactate, pyruvate, and alaninelevels indicate a mitochondrial disorder.	Homozygous for c.1222C > T.	No mention.	He had muscle dystrophy and generalized hypotonia and died at the age of 7 months.
Hoefs et al^[12]^	No mention of age/female	Nonconsanguineous parents.	Nystagmus was detected at 5 mo of age, characterized by failure to thrive, crying, eating difficulties, spasticity, and mental retardation with exacerbations.	MRI at 5 mo was normal. MRI at 16 mo showed hyperintensity on T2 and hypointensity on T1 in subcortical and deep WM, corpus callosum, internal capsule, and brainstem, with restricted diffusion in the affected WM.	No mention.	Compound heterozygous (c.631–633delGAA and c.683T > C).	No mention.	She died at the age of 2 yrs.
Martín et al^[18]^	8-m-old/female	Her brother had a similar clinical presentation and died of respiratory failure at age 8 mo.	Vomiting, floppiness, growth retardation, irritability, horizontal nystagmus, and generalized hypotonia.	MRI showed bilateral lesions affecting the substantial nigra and midbrain.	Increased blood lactate levels.	Homozygous for c.691C > G (p.L231V).	No mention.	Her status worsened 5 mo later when she developed respiratory insufficiency, and she died at age 14 mo.
Kashani et al^[19]^	7-y-old/male	No history of genetic diseases.	Acute neurological deficits at 2 yrs followed by repeated episodes of mild neurological deterioration, subsequent remissions.	MRI showed diffuse cystic leukoencephalopathy with the involvement of the corpus callosum and sparing of the gray matter.	Blood lactate and urinary organic acids were in the normal ranges.	Homozygous for c.755A > G.	No mention.	His cognitive capabilities were at the upper limit of the mild intellectual disability and kept relative health.
Ferreira et al^[20]^	4-y-old/female	Parents were first cousins.	Dystonic posturing of her left arm and loss of motor and language development at the age of 1 yr, followed by hemiparesis, loss of language, and stupor.	Initial MRI showed abnormal signals in the WM; the second MRI showed worsening WM abnormalities with the formation of an increased number of cystic lesions involving WM, corpus callosum, and brainstem.	Urine organic acids and serum and cerebrospinal fluid amino acids were in the normal ranges.	Homozygous forc.1783A > G.	Coenzyme Q10, thiamin, baclofen, and phenobarbital.	She walked on tiptoes and had swallowing difficulties, bowel incontinence, and speech dysfluency at age 4 yrs.
Ferreira et al^[20]^	30-m-old/male	Parents were first cousins.	Reduced facial activity and response to the environment with irritability. Dystonic leg posturing, Babinski sign, reduced interaction, and impaired swallowing at age 13 mo.	MRI showed multiple cystic lesions in the WM, a concentric pattern of abnormalities in FLAIR imaging, and large areas of hypointensity sparing the subcortical WM in T1-WI.	Increased serum lactic acidemia and creatine kinase levels.	A novel homozygous variant: c.1783A > G.	Gastrostomy was performed at age 16 mo.	Follow-up showed that he could sit without support, was growing correctly, and was able to pronounce a few words.

FLAIR = fluid-attenuated inversion recovery, M = month, MRI = magnetic resonance imaging, WI = weighted images, WM = white matter, Y = year.

A retrospective study of 35 patients with LS showed that nearly 1 in 3 patients had predisposing factors, including infection and vaccination.^[21]^ An acute attack of LS can be triggered by infection,^[4,10]^ as shown in this study, or other trauma, including surgery or prolonged fasting.^[11]^

### 4.6. Cell culture and biochemical analysis

Metabolic acidosis and an increased serum or CSF lactate/pyruvate ratio are the main biochemical changes in LS; however, LS cannot be excluded without increased lactate levels in the interictal and early stages of onset, as shown in this case. Most patients with complex I deficiency have elevated blood and CSF lactate levels due to mitochondrial redox dysfunction^[8,21,22]^ and an increased lactate/pyruvate ratio; the lactate/pyruvate ratio exceeded 20 in this patient, suggesting MRC dysfunction.^[23]^ Patients with LS caused by *NDUFS1* mutations also show normal blood lactate and urinary organic acid levels. Measurements of the activities of respiratory chain complexes in skin fibroblasts also contribute to the diagnosis of isolated complex I deficiency. The above-mentioned low residual complex I activity suggests that screening for the *NDUFS1* gene is preferred when patients have very low complex I activity expressed in cultured fibroblasts.^[12]^

### 4.7. Treatment and prognosis

Currently, there are no effective treatments for MC1DN5. Therefore, supplementation with multiple cofactors, such as vitamin B2, vitamin B1, vitamin PP, and coenzyme Q10 may be effective in some cases.^[22]^ Succinate can provide electrons for complex I, vitamin C, and vitamin E to eliminate oxygen-derived free radicals, and L-carnitine and lipoic acid can promote fat and sugar metabolism in mitochondria;^[24]^ they are often used in the treatment of MCID. Maintaining adequate nutrition, moderate aerobic exercise, and supplementation with various cofactors are effective in some cases.^[25]^ Most patients have a poor prognosis^[12,20]^ and die of central respiratory or systemic failure from infection, fatigue, or other inducements.^[22]^ A prenatal diagnosis is valuable for families predisposed to this condition.^[18]^

## 5. Conclusions

In conclusion, LS is characterized by bilateral symmetrical lesions in the basal ganglia and the brainstem. However, MRI can show normal findings in the early stages of LS, and brain lesions and increased lactate levels evolve over time. Lip cyanosis, which can be a precursor of respiratory failure, is one of the first symptoms of LS that requires attention. Repeated respiratory muscle weakness should prompt suspicion of MCID-related LS. Precise genetic analysis is very important for clarifying the diagnosis and treatment, encouraging preliminary testing, and judging the risk of symptom recurrence.

## Author contributions

Writing—original draft, writing—review and editing, validation, and conceptualization.
